# The influence of xylanase on the fermentability, digestibility, and physicochemical properties of insoluble corn-based fiber along the gastrointestinal tract of growing pigs

**DOI:** 10.1093/jas/skab159

**Published:** 2021-05-19

**Authors:** Amy L Petry, Nichole F Huntley, Michael R Bedford, John F Patience

**Affiliations:** 1 Department of Animal Science, Iowa State University, Ames, IA 50011, USA; 2 AB Vista Feed Ingredients, Marlborough, Wiltshire SN8 4AN, UK

**Keywords:** carbohydrase, feed enzyme, hemicellulose, small intestine, swine, viscosity

## Abstract

In theory, supplementing xylanase in corn-based swine diets should improve nutrient and energy digestibility and fiber fermentability, but its efficacy is inconsistent. The experimental objective was to investigate the impact of xylanase on energy and nutrient digestibility, digesta viscosity, and fermentation when pigs are fed a diet high in insoluble fiber (>20% neutral detergent fiber; NDF) and given a 46-d dietary adaptation period. A total of 3 replicates of 20 growing gilts were blocked by initial body weight, individually housed, and assigned to 1 of 4 dietary treatments: a low-fiber control (LF) with 7.5% NDF, a 30% corn bran high-fiber control (HF; 21.9% NDF), HF + 100 mg xylanase/kg (HF + XY [Econase XT 25P; AB Vista, Marlborough, UK]) providing 16,000 birch xylan units/kg; and HF + 50 mg arabinoxylan-oligosaccharide (AXOS) product/kg (HF + AX [XOS 35A; Shandong Longlive Biotechnology, Shandong, China]) providing AXOS with 3–7 degrees of polymerization. Gilts were allowed ad libitum access to fed for 36-d. On d 36, pigs were housed in metabolism crates for a 10-d period, limit fed, and feces were collected. On d 46, pigs were euthanized and ileal, cecal, and colonic digesta were collected. Data were analyzed as a linear mixed model with block and replication as random effects, and treatment as a fixed effect. Compared with LF, HF reduced the apparent ileal digestibility (AID), apparent cecal digestibility (ACED), apparent colonic digestibility (ACOD), and apparent total tract digestibility (ATTD) of dry matter (DM), gross energy (GE), crude protein (CP), acid detergent fiber (ADF), NDF, and hemicellulose (*P* < 0.01). Relative to HF, HF + XY improved the AID of GE, CP, and NDF (*P* < 0.05), and improved the ACED, ACOD, and ATTD of DM, GE, CP, NDF, ADF, and hemicellulose (*P* < 0.05). Among treatments, pigs fed HF had increased hindgut DM disappearance (*P* = 0.031). Relative to HF, HF + XY improved cecal disappearance of DM (162 vs. 98 g; *P* = 0.008) and NDF (44 vs. 13 g; *P* < 0.01). Pigs fed xylanase had a greater proportion of acetate in cecal digesta and butyrate in colonic digesta among treatments (*P* < 0.05). Compared with LF, HF increased ileal, cecal, and colonic viscosity, but HF + XY decreased ileal viscosity compared with HF (*P* < 0.001). In conclusion, increased insoluble corn-based fiber decreases digestibility, reduces cecal fermentation, and increases digesta viscosity, but supplementing xylanase partially mitigated that effect.

## Introduction

Globally, industrial coproducts from the dry and wet milling of cereal grains are often included in swine diets to reduce feed cost; however, they contain greater levels of non-starch polysaccharides (NSP) than their parent grain (Jaworski et al., 2015). It has been well established that pigs lack the enzymes required to digest NSP, and that increased dietary NSP can reduce nutrient and energy digestibility, impair hindgut fermentation, and increase digesta viscosity and rate of passage, resulting in decreased pig performance and carcass yield (Le Goff et al., 2002; Gutierrez et al., 2013; Weber et al., 2015). Supplementing exogenous carbohydrases may aid in ameliorating these negative effects, but their efficacy likely depends on the source, type, structure, and physicochemical properties of the NSP within the diet (Patience and Petry, 2019).

Arabinoxylan is the major constituent of the NSP in industrial coproducts produced from corn (Jaworski et al., 2015). Corn-based arabinoxylans are poorly fermented due to their increased substitution with L-arabinofuranosyl side chains, and poor solubility (Bach Knudsen, 2014). Xylanase, a carbohydrase that hydrolyzes the β-(1–4) glycosidic bonds of arabinoxylan, may improve nutrient and fiber digestibility and subsequent fermentation by degrading arabinoxylan into various mono- and oligosaccharides, releasing trapped nutrients, reducing digesta viscosity, or modulating microbiota to improve fermentation (Adeola, 2011; De Vries et al., 2012; Masey O’Neill et al., 2014). Likewise, there is evidence to suggest supplementing xylanase increases fiber solubility in the ileum (Tiwari et al., 2018), and this soluble NSP fraction in the ileal effluent may subsequently alter digesta viscosity in the large intestine. However, the efficacy of xylanase in pigs fed corn-based diets is variable and not fully understood (Petry and Patience, 2020).

There are contradicting reports on the efficacy of xylanase to improve nutrient and energy digestibility, fermentation, and growth performance in growing pigs (Torres-Pitarch et al., 2019; Petry and Patience, 2020). These inconsistencies could be due to differences in enzyme characteristics, inadequate supplementation time, dietary fiber concentration, or the physicochemical properties of insoluble corn-based fiber (Petry and Patience, 2020). Additionally, results of recent research suggest at least 25 d of adaptation may be needed for xylanase to improve fiber digestibility in the upper small intestine of pigs fed corn-based fiber (Petry et al., 2020a). However, others have reported improvements in ileal and total tract digestibility with shorter adaptation periods (Tsai et al., 2017; Tiwari et al., 2018). The experimental objective was to evaluate the impact of xylanase on nutrient and energy digestibility, nutrient flow, fermentation, and digesta viscosity in the ileum, cecum, colon, and the total tract of growing pigs fed a diet high in insoluble fiber and given a longer adaptation time than what is typically reported. It was hypothesized that when given sufficient adaptation time and substrate, xylanase would improve nutrient flow, nutrient and energy digestibility, fermentation, and alter digesta viscosity of growing pigs fed insoluble corn-based fiber.

## Materials and Methods

All experimental procedures adhered to guidelines for the ethical and humane use of animals for research according to the Guide for the Care and Use of Agricultural Animals in Research and Teaching (FASS, 2010), and were approved by the Iowa State University Institutional Animal Care and Use Committee (#9-17-8613-S).

### Animals, housing, and experimental design

This research is in continuance of a previously published study, and readers are referred to Petry et al. (2020b) for extensive experimental methods. Animal and experimental methods reported are provided to orient readers to the details of the study and treatment design; all analytical methods unique to these data are provided. In brief, a total of 60 gilts with an initial body weight of 25.4 ± 0.9 kg were used in 3 replicates of a 46-d trial. There were 20 gilts per replicate. Pigs were individually housed and allowed ad libitum access to feed for 36 days followed by a 10-d metabolism study. Pigs were blocked by initial body weight and randomly assigned to one of four dietary treatments: a low-fiber control (LF, 7.5% neutral detergent fiber [NDF]); a high-fiber control with 30% corn bran without solubles (HF, 21.9% NDF); HF + 100 mg xylanase/kg (HF + XY [Econase XT 25P; AB Vista, Marlborough, UK]) providing 16,000 birch xylan units/kg; and HF + 50 mg arabinoxylan-oligosaccharide (AXOS) product/kg (HF + AX [XOS 35A; Shandong Longlive Biotechnology, Shandong, China]) providing 48 mg of AXOS with 3–7 degrees of polymerization per kg. The xylanase supplemented in HF + XY was isolated from *Trichoderma reesei* using submerged fermentation. The AXOS supplemented in HF + AX was produced by hydrothermal treatment of corn cobs and xylanase incubation to produce oligosaccharides which range from 3 to 7 degrees of polymerization. The supplemented product contained 95% of AXOS and 5% corn cob powder as a residual carrier. During the metabolism study, all pigs were limit fed 80% of the average daily feed intake among all treatments set by the first experimental replicate, and the daily feed allotment was split into two feedings at 0700 and 1500 hours.

Experimental diets were manufactured in mash form and formulated to meet or exceed the nutritional requirements of growing pigs according to National Research Council (2012), and diets fed during the metabolism period are depicted in Table 1. The level of energy in the diet was allowed to float; equalizing energy in both the low- and high-fiber diets would have required the use of supplemental fat which itself would have confounded the experiment. Avoiding excessive change in diet composition was considered to be a higher priority for the metabolism study, especially because the focus was on nutrient utilization and characteristics of the digesta. Chromium trioxide was included as an indigestible marker to calculate digestibility coefficients as it has similar recovery rates in both high- and low-fiber diets (Jacobs et al., 2017). Diet samples were collected during mixing and stored at −20°C for future analyses.

**Table 1.** Ingredient and nutrient composition (as-fed) of experimental diets fed during metabolism study^1^

Item	Treatment^2^			
	LF	HF	HF + XY	HF + AX
Ingredient composition, %				
Corn	75.996	45.975	45.965	45.970
Corn bran without solubles	0.000	30.000	30.000	30.000
Soybean meal, 46.5%	20.566	20.566	20.566	20.566
Limestone	1.182	1.159	1.159	1.159
Monocalcium phosphate 21%	0.504	0.593	0.593	0.593
Sodium chloride	0.500	0.500	0.500	0.500
Cr_2_O_3_	0.500	0.500	0.500	0.500
L-lysine HCl	0.295	0.267	0.267	0.267
Trace mineral premix^3^	0.200	0.200	0.200	0.200
Vitamin premix^4^	0.140	0.140	0.140	0.140
L-threonine	0.067	0.054	0.054	0.054
DL-methionine	0.043	0.041	0.041	0.041
Phytase^5^	0.005	0.005	0.005	0.005
Xylanase^6^	0.000	0.000	0.010	0.000
AXOS^7^	0.000	0.000	0.000	0.005
Calculated nutrients, %				
Standard ileal digestible lysine, %	0.92	0.92	0.92	0.92
Standard ileal digestible TSAA^8^:lysine	0.56	0.56	0.56	0.56
Standard ileal digestible threonine:lysine	0.61	0.61	0.61	0.61
Standard ileal digestible trpytophan:lysine	0.17	0.17	0.17	0.17
Calcium, %	0.63	0.63	0.63	0.63
Standardized total tract digestible phosphorus, %	0.31	0.31	0.31	0.31
Metabolizable energy Mcal/kg	3.26	3.02	3.02	3.02
Net energy, Mcal/kg	2.46	2.26	2.26	2.26
Analyzed composition, %				
DM	89.12	89.81	89.98	90.15
Starch	41.08	23.55	23.70	23.11
CP	15.56	15.54	15.47	15.60
NDF	7.54	21.91	22.12	22.08
ADF	2.41	5.53	5.78	5.59
aEE^9^	2.48	2.74	2.82	2.89
Cr_2_O_3_	0.49	0.49	0.49	0.49

^1^Adapted from Petry et al. (2020b).

^2^Treatments include: LF, basal corn-soybean diet; HF, basal corn-soybean diet with 30% corn bran at the expense of corn; HF with the addition of xylanase (HF + XY); HF with the addition of AXOS (HF + AX).

^3^Mineral premix provided the following (per kg diet): 165 mg of Fe (ferrous sulfate); 165 mg of Zn (zinc sulfate); 39 mg of Mn (manganese sulfate); 16.5 mg of Cu (copper sulfate); 0.3 mg of I (calcium iodate); 0.3 mg of Se (sodium selenite).

^4^Vitamin premix provided the following (per kg diet): 6,125 IU of vitamin A; 700 IU of vitamin D3; 50 IU of vitamin E; 3 mg of menadione (to provide vitamin K); 11 mg of riboflavin; 27 mg of d-pantothenic acid; 0.05mg of vitamin B12; and 56 mg of niacin.

^5^Quantum Blue 5G, AB Vista, Marlborough, UK.

^6^Econase XT 25P, AB Vista, Marlborough, UK; inclusion level provided 16,000 birch xylan units per kg.

^7^XOS 35A; Shandong Longlive Biotechnology, Shandong, China; 3–7 degrees of polymerization.

^8^TSAA, total sulfur amino acids (Met + Cys).

^9^aEE, acid-hydrolyzed ether extract.

### Sample collections and analytical methods

Fecal samples were collected from d 43 to 46 of the metabolism study and were immediately stored at −20°C for subsequent analysis. On d 46, after the 0700 hours feeding, pigs were randomly selected from across all treatments and euthanized by captive bolt stunning followed by exsanguination. Ileal, cecal, and colonic contents were collected using the slaughter technique. Clamps were place at the stomach to duodenum sphincter, the ileocecal junction, and at the rectum, and the portion of intestines just below the stomach to near the anus were removed from the abdomen. Ileal digesta was collected from a 156 cm section of small intestine measured cranially to the ileocecal junction. Cecal digesta was collected starting at the apex of the cecum until approximately 100 mL of digesta was collected. Colonic digesta collections started at the apex of the spiral colon and moved proximally until the end of the ascending portion of the spiral colon. Digesta samples were snap-frozen in liquid nitrogen and stored at −20°C for subsequent analyses. After the completion of each replicate, fecal samples were thawed, homogenized within pig, and subsampled. Fecal and diet subsamples were dried in a convection oven at 60°C until a constant weight was achieved. Digesta samples were lyophilized. All dried samples were ground through a 1-mm screen using a Wiley Mill (Variable Speed Digital ED-5 Wiley Mill; Thomas Scientific, Swedesboro, NJ). After grinding, all samples were stored in desiccators to maintain dry matter (DM) content.

Diets were analyzed in duplicate for acid-hydrolyzed ether extract (method 2003.06 of AOAC, 2007) and starch (Megazyme total starch assay kit, Wicklow, Ireland; modified method 996.11 of AOAC, 2007). Diet, digesta, and fecal samples were analyzed in duplicate for DM (method 930.15 of AOAC, 2007) and nitrogen (TruMac; LECO Corp., St. Joseph, MI; method 990.03 of AOAC, 2007). An ethylenediaminetetraacetate sample (9.56% nitrogen; determined to have 9.56 ± 0.03% nitrogen) was used for standard calibration and crude protein (CP) was calculated as nitrogen × 6.25. Diets and digesta samples were analyzed in duplicate for chromium trioxide using a modified method of Fenton and Fenton (1979). Relative to Fenton and Fenton (1979), the standard curve was established using feed absent of the marker mixed with 0.00%, 0.10%, 0.20%, 0.40%, 0.80%, 1.60%, 3.20%, or 6.40% chromium trioxide using an analytical scale. The chromium trioxide used in the standard curve was the same product used during diet mixing. The x-axis of the standard curve was defined relative to inclusion rate (%) of chromium trioxide. Diet, digesta, and fecal samples were analyzed in triplicate for NDF (Van Soest and Robertson, 1979) and for acid detergent fiber (ADF; Goering and Van Soest, 1970). The procedures were conducted in succession, and hemicellulose was calculated as NDF minus ADF. The gross energy (GE) of diet, digesta, and fecal samples were determined in duplicate using an isoperibolic bomb calorimeter (model 6200; Parr Instrument Co., Moline, IL). Benzoic acid (6,318 kcal GE/kg; Parr Instrument Co.) was used as the standard for calibration and was determined to contain 6,320 ± 0.94 kcal GE/kg.

The pH of ileal, cecal, and colonic digesta was measured using a portable pH meter (pH 150 Meter Kit, Oakton Instruments, Vernon Hills, IL), and the pH was adjusted for sample temperature using an established temperature curve from the manufacturer. Ileal, cecal, and colonic digesta short-chain fatty acid concentrations were measured via gas chromatograph in triplicate. In brief, wet cecal and colonic digesta (1 g) were diluted with 5 mL of deionized water and mixed overnight on a rocking platform before centrifugation at 20,000 × *g* for 20 min at 4°C. The supernatant (1 mL) was placed into a gas chromatography vial with 0.3 g of NaCl and 100 μL of phosphoric acid. Ileal digesta (2 g) was centrifuged at 20,000 × *g* for 20 min at 4°C before the supernatant (1 mL) was placed into a new tube with 100 μL of phosphoric acid. The tubes were centrifuged at 4,000 × *g* for 10 min at 4°C and the supernatant (1 mL) was placed into a vial with 0.3 g of NaCl. The prepared samples were frozen at −20°C and sent to an external laboratory (USDA-ARS-MWA-NLAE, Ames, IA) for analysis (Agilent 7890A Gas Chromatograph, Agilent Technologies Inc., Wilmington, DE) using methods previously described by Kerr et al. (2015). Digesta SCFA was expressed as the molar proportions of individual SCFA (%) relative to the total SCFA concentration.

The viscosity of ileal, cecal, and colonic whole digesta was measured using a DV rotational viscometer equipped with a V3 vane mixing spindle (Brookfield, Middleboro, MA). Briefly, samples were incubated in a water bath at 90°C for 30 min to cease enzyme activity. Samples were then homogenized by mixing with a spatula and cooled to 37°C in a water bath. Approximately 30 mL of digesta were placed in 100 mL glass beakers with a diameter of 5.2 cm and measured at 0.5, 1, 2, 4, 10, and 20 revolutions/min. Sample temperature was maintained at 37°C ± 0.5 to simulate a physiologically relevant impact of temperature on viscosity. Whole intestinal contents exhibit non-Newtonian shear-thinning behavior in that when the revolutions per minute of the spindle increases the viscosity of the sample decreases exponentially. Thus, measuring viscosity at one shear rate, or averaging values, can inadequately portray the rheological properties of the sample. To account for this property, a viscosity curve was established for each sample, and a viscosity value for a given sample was defined as the intercept of the exponential relationship in Pa⋅s.

### Calculations

The apparent ileal digestibility (AID), apparent cecal digestibility (ACED), apparent colonic digestibility (ACOD), and apparent total tract digestibility (ATTD) of DM, GE, CP, NDF, ADF, and hemicellulose were calculated according to the equation of Oresanya et al. (2007):

AID, ACED, ACOD, or ATTD% = {100 − [100 × (% Cr_2_O_3_ in feed/% Cr_2_O_3_ in ileal, cecal, and colonic digesta or feces) × (concentration of component in ileal, cecal, and colonic digesta or feces/concentration of component in feed)]}.

The flow of DM (DM intake g/d) or NDF (g/d of NDF intake) within the digestive tract were calculated using the methodology of Weiland (2017), and expressed on a weight and proportion of intake basis:

Disappearance prior to the terminal ileum (TI) = {intake of component − amount remaining at TI [calculated as concentration of component in digesta × (Cr_2_O_3_ in diet/Cr_2_O_3_ in digesta)]}.

Hindgut disappearance = {amount remaining at TI − amount excreted in feces [calculated as concentration of component in feces × (Cr_2_O_3_ in diet/Cr_2_O_3_ in feces)]}.

Cecal disappearance = [(ACED% of a given component × intake of component) − disappearance prior to the TI].

Colonic disappearance = [(ATTD% of a given component × intake of component) − (disappearance prior to the TI + cecal disappearance)].

### Statistical analysis

Data were analyzed according to the following mixed model using PROC MIXED in SAS 9.3 (SAS Inst., Cary, NC):


$$\begin{array}{l} Y_{ijkl}=\mu +\tau_{i}+\upsilon_{j}+\rho_{k}+e_{ijkl}, \end{array}$$

where *Y*_*ijkl*_ is the observed value for *l*th experimental unit within the *i*th level of dietary treatment of the *j*th block for the *l*th pig in the *k*th replicate; *μ* is the general mean; *τ*_*i*_ is the fixed effect of the *i*th diet (*i* = 1–4); *υ*_*j*_ is the random effect of the *j*th block (*j* = 1–5); *ρ*_*k*_ is the random effect of the *k*th replicate (*k* = 1–3); and *e*_*ijkl*_ is the associated variance as described by the model for *Y*_*ijkl*_ (*l* = 1 through 60); assuming $\upsilon_{j}\;∼N\left(0,\;I\sigma_{\upsilon_{j}}^{2}\right),\;\rho_{k}\;∼N(0,\;I\sigma_{\rho_{k\;}}^{2}$, and $e_{ijkl}∼N(0,\;I\sigma_{e}^{2})$, where *I* is the identity matrix.

The normality and homogeneity of the studentized residuals from the reported model were verified. Outliers were removed if studentized residuals were greater than three standard deviations away from the mean residual. Least square means were separated using Fisher’s least significant difference test, and treatment differences were considered significant if *P* ≤ 0.05 and trends if 0.05 > *P* < 0.10.

## Results

All gilts completed the experiment, and their performance data were reported in Petry et al. (2020b). Diarrhea was observed in a few gilts in replicates 2 and 3 during the adaptation period, but no diarrhea was observed during the metabolism crate period. All gilts within replicates 2 and 3 during the adaptation period were treated with tylosin phosphate according to label instructions. No interaction between treatment and replicate was observed for the reported dependent variables, and thus the effect of replicate was implemented as a random effect.

### Physicochemical properties of digesta

**Figure 1.**
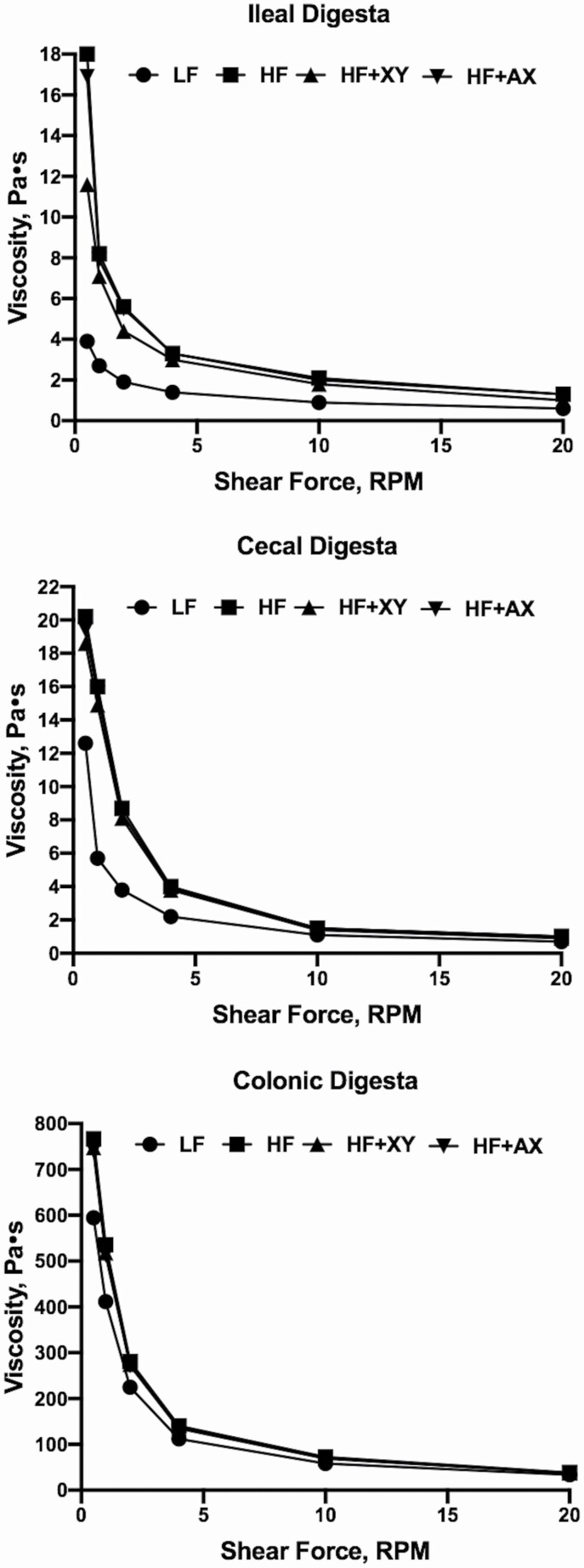
The relationship between measured viscosity at a given shear rate for ileal, cecal, and colonic digesta.

Irrespective of collection site, digesta viscosity displayed shear-thinning behavior in that as shear force increased, digesta viscosity decreased (Figure 1). As expected, the more distal the collection site, relative to the ileum, the greater the digesta viscosity. Compared with LF, HF increased ileal viscosity (9.7 vs. 2.7 Pa∙s; *P* < 0.01) but the addition of xylanase partially mitigated that effect (Table 2; *P* < 0.001). In both the cecum and proximal colon, LF had lower digesta viscosity (*P* < 0.001). Pigs fed HF and HF + AX had increased ileal pH relative to LF and HF + XY (*P* = 0.001). However, cecal and colonic pH did not differ among treatments (*P* = 0.193 and *P* = 0.399, respectively).

**Table 2.** Effect of dietary treatment on digesta viscosity and pH^1^

	Diet^2^				SEM	*P-*value
Item	LF	HF	HF+XY	HF+AX		
Digesta viscosity^3^, Pa▪s						
Ileal	2.7^a^	9.7^b^	7.2^c^	9.3^b^	0.4	<0.001
Cecal	7.3^a^	13.5^b^	12.5^b^	13.1^b^	0.7	<0.001
Colonic	372.1^a^	478.6^b^	465.4^b^	460.7^b^	13.8	<0.001
Digesta pH						
Ileal	6.79^a^	7.29^b^	6.68^a^	7.11^b^	0.25	0.001
Cecal	5.93	6.02	6.07	6.10	0.34	0.193
Colonic	6.39	6.38	6.34	6.52	0.30	0.399

^1^ *n* = 15 pigs per treatment per treatment-fed experimental diets for a 36-d adaptation period followed by a 10-d metabolism crate study.

^2^Dietary treatments include: LF, basal corn-soybean diet; HF, basal corn-soybean diet with 30% corn bran at the expense of corn; HF with the addition of xylanase (HF + XY); HF with the addition of AXOS (HF + AX).

^3^Viscosity values are the intercept of the exponential relationship of the observed shear-thinning behavior for a given sample measured a six shear force rates.

^a,b,c^Within a row, means without a common superscript differ (*P* ≤ 0.05).

### Fermentation

**Figure 2.**
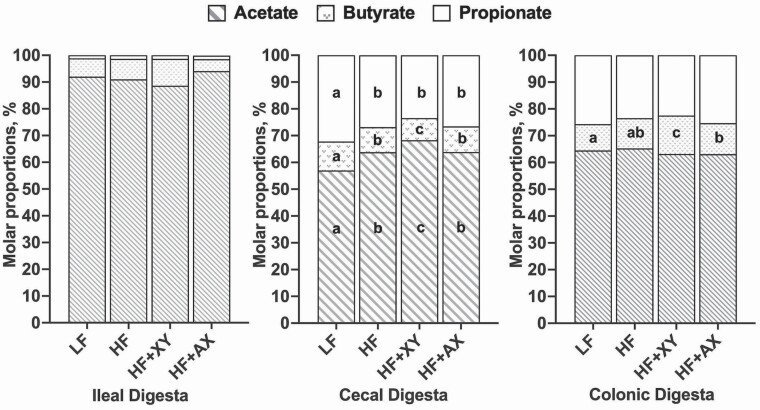
The molar proportion of short-chain fatty acids among treatments in ileal, cecal, and colonic digesta. Letters within a section of a bar denote statistically differences (*P* < 0.05) for a given SCFA measured in a given location.

The molar proportions of SCFA in ileal digesta did not differ among treatments (Figure 2; *P* > 0.05). Among treatments, LF had the greatest proportion of propionate in the cecal digesta (*P* < 0.05). Likewise, LF had the greatest proportion of butyrate in the cecum, and HF + XY had the lowest (*P* < 0.05). Among all treatments, pigs fed xylanase had the greatest molar proportion of acetate in the cecum and butyrate in the colon (*P* < 0.05).

### Nutrient and energy digestibility

Compared with LF, HF reduced the AID of DM (74.81 vs. 56.48%; *P* < 0.01), but xylanase partially mitigated this effect and had increased AID of DM relative to HF (Table 3; *P* < 0.001). Similarly, when compared with LF, HF reduced the AID of GE, but HF + XY performed intermediately between the two (*P* < 0.001). The AID of CP was lower in pigs fed HF, relative to LF (*P* < 0.01), but pigs fed HF + XY and HF + AX had greater AID of CP relative to HF, respectively (*P* < 0.05). Likewise, compared with LF, HF reduced the AID of NDF, but HF + XY and HF + AX had AID of NDF values intermediately between the controls (*P* < 0.001). Pigs fed HF + XY had the greatest AID of ADF among treatments (*P* = 0.023). The AID of hemicellulose was greatest in LF (*P* < 0.01). However, compared with HF, the addition of xylanase improved the AID of hemicellulose (28.26 vs. 23.97; *P* < 0.001).

**Table 3.** Effect of treatment on the AID, ACED, ACOD, and ATTD, of DM, GE, CP, NDF, ADF, and hemicellulose^1^

	Diet^2^				SEM	*P-*value
Item^3^	LF	HF	HF+XY	HF+AX		
AID, %						
DM	74.81^a^	56.48^b^	62.38^c^	57.28^b^	1.25	<0.001
GE	80.79^a^	61.04^b^	65.69^c^	62.41^b^	1.24	<0.001
CP	80.79^a^	75.25^b^	78.52^c^	76.80^c^	1.28	<0.001
NDF	30.82^a^	20.51^b^	25.76^c^	23.85^c^	2.82	<0.001
ADF	19.01^a^	16.82^b^	22.13^c^	17.78^b^	2.23	0.023
Hemicellulose	36.21^a^	23.97^b^	28.26^c^	23.81^b^	2.76	<0.001
ACED, %						
DM	80.23^a^	63.96^b^	68.97^c^	64.59^b^	0.91	<0.001
GE	81.30^a^	66.43^b^	70.99^c^	66.69^b^	0.93	<0.001
CP	81.57^a^	78.24^b^	81.71^a^	78.83^b^	0.78	<0.001
NDF	32.52^a^	22.74^b^	32.19^a^	23.25^b^	2.89	<0.001
ADF	21.60^a^	17.14^b^	26.15^c^	19.14^b^	2.84	<0.001
Hemicellulose	37.70^a^	24.59^b^	34.33^c^	25.63^b^	2.70	<0.001
ACOD, %						
DM	88.68^a^	74.13^b^	75.93^c^	73.80^b^	0.58	<0.001
GE	89.30^a^	75.56^b^	77.01^c^	75.19^b^	0.58	<0.001
CP	86.83^a^	81.71^b^	83.43^c^	80.78^b^	0.71	<0.001
NDF	63.05^a^	40.43^b^	46.34^c^	39.10^b^	2.18	<0.001
ADF	51.22^a^	40.12^b^	45.44^c^	39.16	2.55	<0.001
Hemicellulose	62.10^a^	40.53^b^	43.27^c^	39.08	2.87	<0.001
ATTD, %						
DM	88.73^a^	75.59^b^	78.20^c^	74.35^b^	0.67	<0.001
GE	89.01^a^	77.40^b^	79.13^c^	76.23^b^	0.88	<0.001
CP	87.16^a^	81.77^b^	84.22^c^	80.91^b^	0.77	<0.001
NDF	63.32^a^	44.44^b^	53.86^c^	42.72^b^	2.94	<0.001
ADF	59.12^a^	42.86^b^	52.07^c^	40.19^b^	2.94	<0.001
Hemicellulose	64.77^a^	44.68^b^	53.40^c^	43.46^b^	2.69	<0.001

^1^ *n* = 15 pigs per treatment per treatment-fed experimental diets for a 36-d adaptation period followed by a 10-d metabolism crate study.

^2^Dietary treatments include: LF, basal corn-soybean diet; HF, basal corn-soybean diet with 30% corn bran at the expense of corn; HF with the addition of xylanase (HF + XY); HF with the addition of AXOS (HF + AX).

^3^AID, ACED, ACOD, and ATTD% = {100 − [100 × (% Cr_2_O_3_ in feed/% Cr_2_O_3_ in ileal, cecal, and colonic digesta or feces) × (concentration of component in ileal, cecal, and colonic digesta or feces/concentration of component in feed)]}.

^a,b,c^Within a row, means without a common superscript differ (*P* ≤ 0.05).

When compared with LF, HF reduced the ACED of DM, GE, CP, NDF, ADF, and hemicellulose, respectively (*P* < 0.01). However, relative to HF, the addition of xylanase improved the ACED of DM, GE, CP, NDF, and hemicellulose respectively (*P* < 0.05). Pigs fed HF + XY had the greatest ACED of ADF (*P* < 0.05). The ACED of DM, GE, CP, NDF, ADF, and hemicellulose of pigs fed HF + AX did not differ from HF (*P* > 0.05). Relative to LF, HF reduced the ACOD of DM, GE, CP, NDF, ADF, and hemicellulose respectively (*P* < 0.01). Xylanase partially mitigated this impact, improving the ACOD of DM, GE, CP, NDF, and hemicellulose, relative to HF, respectively (*P* < 0.05). The ATTD of DM, GE, CP, NDF, ADF, and hemicellulose was greater in pigs fed LF, relative to HF, respectively (*P* < 0.001). However, the ATTD of DM, GE, CP, NDF, ADF, and hemicellulose in pigs fed HF + XY was greater than HF, respectively (*P* < 0.05). The ATTD of DM, GE, CP, NDF, ADF, and hemicellulose of pigs fed HF + AX did not differ from HF (*P* > 0.05).

### DM and NDF flow

By design, DM intake was similar among treatments during the time pigs were housed in metabolism crates, and NDF intake was greater in the high-fiber treatments (Table 4). Pigs fed LF had the greatest disappearance of DM in the small intestine, and pigs fed HF + XY digested 80 g more DM in the small intestine than HF (*P* = 0.006). Interestingly, HF had the greatest hindgut disappearance of DM among treatments, whereas LF had the lowest (*P* = 0.031). This can be attributed to the increase in post-cecal DM disappearance observed in HF (*P* = 0.034). However, pigs fed HF + XY had greater cecal disappearance of DM and NDF (*P* = 0.008 and *P* < 0.001, respectively). Relative to HF, the addition of xylanase improved the fermentation of DM and NDF in the cecum, respectively (*P* < 0.05). When expressed on a weight basis, LF had lower post-cecal disappearance of NDF relative to HF (*P* < 0.05), but when expressed as a proportion of NDF intake, LF had a greater proportion of its NDF intake digested post the cecum (*P* < 0.05). Pigs fed HF excreted more NDF and DM when compared with LF (*P* < 0.01). However, xylanase partially mitigated this effect, reducing DM and NDF excretion, relative to HF, respectively (*P* < 0.05).

**Table 4.** The influence of treatment on NDF and DM flow within the digestive tract and proportion of NDF disappearance within in the small intestine, cecum, and post cecum^1^

	Diet^2^				SEM	*P-*value
Item	LF	HF	HF + XY	HF + AX		
NDF intake, g/d	157	456	460	459	–	–
Disappearance prior to TI^3^, g	43^a^	93^b^	105^c^	100^c^	4	<0.001
Hindgut disappearance^4^, g	65^a^	118^b^	135^c^	94^b^	17	0.027
Cecal disappearance^5^, g	6^a^	13^a^	44^b^	8^a^	7	<0.001
Colonic disappearance^6^, g	59^a^	104^b^	92^b^	86^b^	12	<0.001
Excreted in feces^7^, g	48^a^	245^b^	220^c^	257^b^	14	<0.001
Proportion of NDF intake, %						
Disappeared prior to TI	27.6^a^	20.4^b^	22.7^c^	23.7^c^	2.2	0.012
Disappeared in cecum	4.1^a^	3.0^a^	9.5^b^	1.8^c^	2.4	<0.001
Disappeared post cecum	37.6^a^	22.8^b^	19.9^c^	18.6^c^	2.3	0.044
Excreted in feces	30.7^a^	53.8^b^	47.9^c^	55.9^b^	5.6	<0.001
DM intake g/d	1,854	1,868	1,872	1,875	–	–
Disappearance prior to TI, g	1,382^a^	1,088^b^	1,168^c^	1,104^b^	28	0.006
Hindgut disappearance, g	263^a^	324^b^	301^c^	291^c^	16	0.031
Cecal disappearance, g	103^a^	98^a^	162^c^	96^a^	10	0.008
Colonic disappearance, g	160^a^	226^b^	139^a^	195^b^	29	0.034
Excreted in feces, g	209^a^	457^b^	403^c^	473^b^	22	0.009
Proportion of DM intake, %						
Disappeared prior to TI	74.6^a^	58.2^b^	62.4^c^	59.3^b^	1.6	0.014
Disappeared in cecum	5.5^a^	5.2^a^	8.7^b^	5.1^a^	0.9	0.015
Disappeared post cecum	8.6^a^	12.1^b^	7.4^a^	10.4^b^	1.4	0.018
Excreted in feces	11.3^a^	24.4^b^	21.5^c^	25.2^b^	2.1	0.017

^1^ *n* = 15 pigs per treatment per treatment-fed experimental diets for a 36-d adaptation period followed by a 10-d metabolism crate study.

^2^Dietary treatments include: LF, basal corn-soybean diet; HF, basal corn-soybean diet with 30% corn bran at the expense of corn; HF with the addition of xylanase (HF + XY); HF with the addition of AXOS (HF + AX).

^3^Disappearance prior to the TI = [intake of component − amount remaining at TI (calculated as concentration of component in digesta × (Cr_2_O_3_ in diet/Cr_2_O_3_ in digesta)].

^4^Hindgut disappearance = (amount remaining at TI − amount excreted in feces).

^5^Cecal disappearance = [(ACED% of a given component × intake of component) − disappearance prior to the TI].

^6^Colonic disappearance = [(ATTD% of a given component × intake of component) − (disappearance prior to the TI + cecal disappearance)].

^7^Excreted in feces= [concentration of component in feces × (Cr_2_O_3_ in diet/Cr_2_O_3_ in feces)].

^a,b,c^Within a row, means without a common superscript differ (*P* ≤ 0.05).

## Discussion

Swine nutritionists will often formulate diets with industrial coproducts to reduce feed cost, but this frequently increases dietary NSP concentrations and lowers dietary starch (Acosta et al., 2020). Indeed, HF contained almost three times the amount of NDF compared to LF, and 42.7% less starch. Pigs are poor utilizers of NSP and increasing its concentration will decrease nutrient and energy digestibility (Acosta et al., 2020), reduce relative hindgut fermentation (Gutierrez et al., 2013), impair pig performance and carcass yield (Beaulieu et al., 2009), and dilute dietary energy (Gutierrez et al., 2013). Thus, it is logical that a diet with 30% corn bran without solubles, an industrial coproduct high in NSP, markedly decreased nutrient and energy digestibility along the gastrointestinal tract. This is in agreement with others who utilized corn-based industrial coproducts (Gutierrez et al., 2013; Acosta et al., 2020). Carbohydrases can partially ameliorate these antinutritive effects of NSP potentially resulting in improved nutrient digestibility, increasing NSP fermentation, and mitigating certain negative physicochemical properties (Bedford, 2018).

Xylanase, an enzyme that hydrolyzes the β-(1–4) glycosidic bonds of arabinoxylan, is often supplemented in corn-based diets to mitigate the antinutritive effects of NSP. This enzyme is consistently effective in poultry fed insoluble corn-based fiber (Raza et al., 2019), but in swine, studies often show no improvements in growth performance, and inconsistent improvements in nutrient and energy digestibility (Abelilla and Stein, 2019; Petry et al., 2019; Torres-Pitarch et al., 2019). Numerous explanations have been proposed for these inconsistencies; length of supplementation, fiber type, and NSP concentration are among the most commonly cited (Petry and Patience, 2020). Digestibility studies evaluating xylanase often use short adaptation periods, less than 10 days, and NDF concentrations less than 18% (Abelilla and Stein, 2019; Torres-Pitarch et al., 2019). As such, one objective of this study was to evaluate the impact of xylanase in growing pigs fed a diet higher in insoluble fiber when the pigs are given a longer adaptation time than what is typically used. Xylanase efficacy in this study may be a consequence of the longer adaptation time, but this was not directly evaluated. Xylanase has been shown to improve NDF digestibility in the upper small intestine and growth performance with increasing supplementation time in growing pigs fed insoluble corn-based fiber (Petry et al., 2020a, 2020b).

The improvements in the AID of energy and nutrients observed in HF + XY are in agreement with Passos et al. (2015) who reported a linear improvement in the AID of DM, GE, and NDF in growing barrows fed a corn–soybean meal-based diet with increasing xylanase supplementation, and with Tiwari et al. (2018), who found improvements in the AID of GE and NSP in nursery pigs fed corn-based diets with xylanase. However, others have reported no improvement in the AID of similar dietary components with xylanase supplementation in corn-based diets (Abelilla and Stein, 2019). Several mechanisms of action for how xylanase could improve nutrient and energy digestibility have been proposed. Logically, xylanase may increase fiber digestibility through arabinoxylan hydrolysis (Tiwari et al., 2018), it may partially mitigate the nutrient encapsulation effect of NSP (Le et al., 2013), or ameliorate its physicochemical properties that negatively impact nutrient digestibility (De Vries et al., 2012).

It is likely that xylanase is hydrolyzing the arabinoxylan structure into smaller fragments that can be directly utilized by the pig (i.e., xylose; Huntley and Patience, 2018) or fermented in the distal ileum (i.e., AXOS), with the AXOS being produced at a higher concentration (Pedersen et al., 2015). There is both in vitro and in vivo evidence suggesting xylanase releases AXOS from wheat (Pedersen et al., 2015; Ravn et al., 2016). Moreover, in corn-based diets, xylanase increases the production of soluble NSP in ileal digesta, and this is likely a result of increased soluble AXOS production (Tiwari et al., 2018). Xylanase-produced AXOS can be fermented by gastrointestinal microbiota producing SCFA that can be utilized by the pig. These data suggest that fermentation may start in the distal ileum, as evidenced by the reduction in ileal pH observed in HF + XY, but it was not substantial enough to alter SCFA composition. Potentially, xylanase did increase ileal SCFA composition, but those SCFA were rapidly absorbed, and not captured in the SCFA analysis of digesta at one point in time. The greater molar proportion of acetate in the cecal digesta, ACED of NDF, and cecal disappearance of NDF, suggest xylanase improves cecal fermentation by providing a more favorable substrate in the ileal effluent, such as soluble AXOS. However, directly supplementing AXOS in this study did not elicit a similar response to xylanase. This is contradictory to when AXOS is directly supplemented in poultry diets (Morgan et al., 2019; Bautil et al., 2020). Although poultry and swine are both monogastrics, there are key differences in their gastrointestinal anatomy, particularly the length of their small intestine relative to their body weight (Moran, 1982). Potentially, the AXOS supplemented in HF + AX was largely fermented in the distal ileum, and thus, unable to modulate digestibility in the hindgut. Supplementing xylanase directly may produce a greater ileal AXOS concentration than what was supplemented in HF + AX and would continue to produce AXOS throughout the gastrointestinal tract (Pedersen et al., 2015).

Xylanase-produced AXOS may act in a stimbiotic manner by modulating intestinal microbiota, increasing microbial diversity, and upregulating fiber-degrading microbial communities that will increase the ability of the large intestine to ferment fiber (Bedford, 2018; Zhang et al., 2018). A stimbiotic, as defined by González-Ortiz et al. (2019), is an additive that stimulates a fiber-degrading microbiome resulting in an increase in fiber fermentability even though the additive itself contributes little to SCFA production. Indeed, the shift in SCFA composition and increased ACED of NDF may suggest xylanase modulates intestinal microbiota to favor fiber fermentation. Acetate is a major fermentation product of soluble xylan degradation in the gastrointestinal tract of humans (Macfarlane and Macfarlane, 2006). There is a paucity of studies evaluating the impact of xylanase on the composition of SCFA in digesta of pigs, and a change in composition may not be indicative of increased SCFA production. However, in vitro fermentation of corn dried distiller grain without solubles with xylanase resulted in an increase in acetate production (Tiwari et al., 2018). Likewise, work by Raven et al. (2016) found increased butyrate production from in vitro fermentation of AXOS produced from xylanase hydrolysis of insoluble wheat bran. Xylanase may upregulate butyrate production indirectly via the metabolism of acetate by *Bifidobacterium* (Feng et al., 2018). The modulation of acetate and butyrate production in the hindgut may partially explain unexpected health benefits of xylanase, such as reduced mortality and increased markers of gut health (Zier-Rush et al., 2016; Duarte et al., 2019). Butyrate is the preferential energy source for colonocytes and can impact colonic health and gut barrier integrity through mediation of the nuclear NF-kB pathway and histone deacetylase inhibition (Bach Knudsen et al., 2018). Likewise, acetate can interact with G-protein coupled FFAR2 receptors to activate the NLRP3 inflammasome pathway, which is known to modulate intestinal epithelial integrity, repair, and homeostasis (Macia et al., 2015).

The impact of xylanase on CP and ADF digestibility observed may be better explained by the mitigation of the nutrient encapsulation effect of NSP (Le et al., 2013). There is in vitro evidence to suggest xylanase can disrupt the structural integrity of plant cell walls through arabinoxylan degradation (Jha et al., 2015). As a result of this disruption, it is plausible that stored starch and protein granules are exposed to endogenous enzymes in the small intestine and to hindgut microbiota. This disruption of the plant cell wall could theoretically improve the access of cellulolytic bacteria to cellulose, which would support the improvement in ADF digestibility observed, and by Tsai et al. (2017). Arabinoxylans aid in connecting the primary and secondary cell wall of monocots, such as corn. This primary cell wall is composed of predominately cellulose microfibrils, and they are anchored in the middle laminae and secondary cell walls by arabinoxylans (Santiago et al., 2013). This creates a complex structure that helps to rigidify the cell wall architecture of mature corn but reduces the exposed surface area of cellulose (Akin and Rigsby, 2008). Possibly, the degradation of arabinoxylan by xylanase disturbs this complex structure and increases the surface area of cellulose to cellulolytic bacteria. On the other hand, the improvements in ADF digestibility may also be explained by the release of soluble feruloylated AXOS by xylanase, which has been observed in vitro (Malunga and Beta, 2016). Corn arabinoxylan is highly substituted with ferulic acid, and that phenolic compound is captured in the ADF fraction of detergent fiber analysis as a lignin constituent (Jung and Fahey, 1986). Potentially, if xylanase releases soluble feruloylated AXOS in vivo, this may also indirectly improve ADF digestibility, as soluble AXOS are not captured by this fiber methodology.

In poultry, supplementing xylanase commonly improves the digestibility of certain cereal grains by decreasing digesta viscosity (Masey O’Neill et al., 2014). Soluble high molecular weight arabinoxylans increase the viscosity of digesta and reduce nutrient digestibility by forming gels that prevent the interaction of endogenous enzymes with nutrients (Dikeman and Fahey, 2006). However, corn-based fiber is highly insoluble in nature and does not form viscous gels. In contrast, it could be reasonably hypothesized that if xylanase increases the concentration of soluble NSP, like Tiwari et al. (2018) observed, then it could potentially increase the viscous nature of digesta. However, in this study opposing results were observed, because xylanase reduced ileal digesta viscosity. The impact of xylanase on digesta viscosity in pigs, irrespective of cereal type, is quite variable (Willamil et al., 2012; Passos et al., 2015; Tiwari et al., 2018). This variability may be a function of the different characteristics of the xylanase sources used in these studies, or an artifact of the method utilized for measuring viscosity. Intestinal digesta expresses pseudoplastic shear-thinning behavior, in that as shear rate increases, viscosity decreases (Dikeman and Fahey, 2006). As such, measuring digesta viscosity at one shear rate may not adequately explain the rheological nature of digesta, or the influence of xylanase on viscosity. Likewise, measuring the viscosity of digesta supernatant is not directly indicative of the rheological properties of whole digesta (Dikeman et al., 2007). Increasing dietary NDF through the addition of corn bran without solubles increased digesta viscosity across the gastrointestinal tract. This is likely a function of increased digesta DM, as it has been shown that increased AID of DM decreases digesta viscosity when measured on whole digesta (Dikeman et al., 2006). This may explain the decreased ileal viscosity observed in LF and HF + XY.

## Conclusions

Increasing dietary NDF by adding corn bran without solubles decreased energy and nutrient digestibility, altered the flow of DM and NDF, and increased digesta viscosity. Supplementing xylanase, but not AXOS, partially mitigated the impact of increased corn-based fiber on energy and nutrient digestibility and fermentation. This is likely occurring by mitigating the nutrient encapsulation effect of NSP and producing soluble AXOS which can be fermented by intestinal microbiota and act as a stimbiotic to favor fiber fermentation. However, the impact of xylanase on digesta viscosity may be limited to the small intestine, and it appears xylanase does not impact digesta viscosity in the large intestine, where xylanase’s efficacy to improve digestibility was greatest.

## Abbreviations

ACED: apparent cecal digestibility
ACOD: apparent colonic digestibility
ADF: acid detergent fiber
AID: apparent ileal digestibility
ATTD: apparent total tract digestibility
AXOS: arabinoxylan oligosaccharides
CP: crude protein
DM: dry matter
GE: gross energy
NDF: neutral detergent fiber
NSP: non-starch polysaccharides
SCFA: short-chain fatty acids
